# Structure Analysis and Study of Biological Activities of Condensed Tannins from *Bruguiera gymnorhiza* (L.) Lam and Their Effect on Fresh-Cut Lotus Roots

**DOI:** 10.3390/molecules26051369

**Published:** 2021-03-04

**Authors:** Xuelian Liu, Ting Chen, Qin Wang, Jiaai Liu, Yuhao Lu, Yan Shi

**Affiliations:** 1 School of Life Sciences, Xiamen University, Xiamen 361102, China; liuxuelian921118@163.com (X.L.); tchen@stu.xmu.edu.cn (T.C.); qwang@xmu.edu.cn (Q.W.); 21620181153698@stu.xmu.edu.cn (J.L.); 21620172203374@stu.xmu.edu.cn (Y.L.); 2 National Experiment Teaching Demonstration Center of Life Sciences, Xiamen University, Xiamen 361102, China

**Keywords:** condensed tannins, structure analysis, biological activities, fresh-cut lotus root, fresh-keeping

## Abstract

*Bruguiera gymnorhiza* (L.) Lam is a mangrove plant that spread in many parts of the world. Though mangrove plant polyphenols have been reported to exhibit many biological activities, little is known about mangrove plant tannins. To explore the application value of tannins from *B. gymnorhiza*, analyses on the structure and biological activity of condensed tannins (CTs) from *Bruguiera gymnorhiza* (L.) Lam were carried out. The results from ^13^C nuclear magnetic resonance (^13^C-NMR) and reversed-phase, high-performance liquid chromatography (RP-HPLC) showed that the CTs were dominated by procyanidins, with a small quantity of prodelphinidins and propelargonidins; and that the monomeric constituents of *B. gymnorhiza* tannins were catechin/epicatechin, gallocatechin/epigallocatechin and afzelechin/epiafzelechin. The CTs were reversible and mixed competitive inhibitors of tyrosinase and the 50% inhibiting concentration (IC_50_) was estimated to be 123.90 ± 0.140 μg/mL. The antioxidant activities of CTs from *B. gymnorhiza* leaves were evaluated, the IC_50_ for 2,2-diphenyl-1-picrylhydrazyl (DPPH) and 2,2′-azino-bis (3-ethylbenzo-thiazoline-6-sulfonic acid diammonium salt) (ABTS) scavenging activities were 88.81 ± 0.135 and 105.03 ± 0.130 μg/mL, respectively, and the ferric ion reducing antioxidant power (FRAP) value was 1052.27 ± 4.17 mgAAE/g. In addition, the results from fresh-keeping assays on fresh-cut lotus root reveal that CTs from *B. gymnorhiza* had excellent effects on inhibiting the activities of polyphenol oxidase (PPO) and peroxidase (POD), protecting fresh-cut lotus root from the oxidation of total phenolics and malondialdehyde (MDA) content and slowing the increase in total phenol content (TPC) at 4 °C during the whole storage period. Therefore, CTs showed good effects against the browning of fresh-cut lotus root. Together, these results suggested that *B. gymnorhiza* CTs are promising antibrowning agents for fresh-cut fruits.

## 1. Introduction

Tyrosinase (EC 1.14.18.1) is a multifunctional, copper-containing oxidase widely distributed in animals (called TYR) and plants (called PPO). It is a key enzyme for the catalyzation of melanin production and insect defense mechanisms, playing an important role in wound healing, hardening and molting. Currently, insecticides have been developed to inhibit TYR activity [1]. Tyrosinase also plays a key role in enzyme-catalyzed browning reactions in fruits and vegetables, affecting the appearance and nutritional quality of food. It is generally believed that tyrosinase inhibitor can inhibit excessive enzyme activity in foods and can also scavenge free radicals and protect the skin from ultraviolet radiation [2].

*B. gymnorhiza* is one of the four dominant species in the mangrove family in China, widely distributed along the coast of Guangxi, Shandong, Hainan, Fujian and Zhejiang. Internationally, they are mainly distributed in Southeast Asia, Australia, South and East Africa and Polynesia [3]. In Thailand, the fruits and flowers of *B. gymnorhiza* are often used in cooking food [4]. The variation between species has been analyzed by PCR-RFLP, indicating a low intraspecific variation in mangrove [5] as a natural, intraspecific, hybrid chloroplast donor [6]. Recent studies showed that *B. gymnorhiza* not only has wood products function, but also has many biological functions, such as antipyretic, antidiarrheal, anti-inflammatory, bactericidal [7] and insecticidal effects [8]. The roots, leaves and fruits of the *B. gymnorhiza* are used in the treatment of diarrhea [9] and burns.

**Figure 1:**
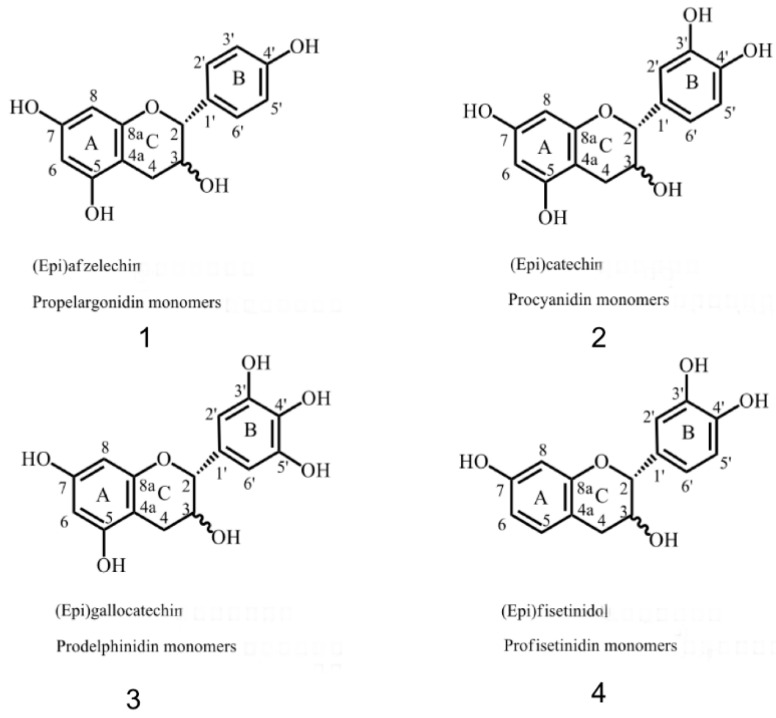
Type of condensed tannins (CTs) [14]. Annotation: Flavane-3-ol monomer is the basic structure of CTS, which can be divided into (**1**) propelargonidin (PP), (**2**) procyanidins (PC), (**3**) prodelphinidins (PD) and (**4**) profisetinidin according to the structural differences of the flavane-3-ol.

Gallotannins, ellagitannins, complex tannins and condensed tannins (CTs) are the four categories of tannins. Chemically, CTs are known as polymerized flavonoids. CTs are oligomeric or polymeric and are formed by C4 of one catechin combined with C8 or C6 of another catechin, and are also polymers formed by the condensation of flavan-3-ol subunits, including propelargonidin (PP), procyanidins (PCs) and prodelphinidins (PDs), as shown in Figure 1. The corresponding basic structural units are catechin/epicatechin, gallocatechin/epigallocatechin and afzelechin/epiafzelechin. Proanthocyanidins (PAs) are the most common in nature, composed of epicatechin. Different connections present different CTs, with typical proanthocyanidine CTs possessing unsubstituted catechin units, such as B-type proanthocyanidins (mainly) linkages (C4-C6/C4-C8) and A-type proanthocyanidins linkages (linkage between C2 and C7). In theory, the degree of polymerization of CTs could reach more than 50 monomer units. The degree of polymerization between CTs differs extensively, with oligomeric CTs consisting of two to ten catechin and epicatechin units [10]. Therefore, the isolation and purification of CTs and their structure determination are challenging. The structural diversity of PAs depends on the diversity of the monomer units, such as interflavane bond polymerization, linkage type and modification of 3-hydroxyl [11] substituents, which all increase the diversity of CT functions, including anti-α-glucoidase [12], anticancer [13], antityrosinase and other activities. CTs are compounds with potential activity against diabetes, skin diseases and inflammation-related diseases. The application of CTs in the pharmaceutical industry is currently a research hotspot.

The demand for fresh fruit continues to grow at a time when people are becoming increasingly concerned about nutrition and healthy lifestyles [15]. To meet consumers’ demand for healthy and convenient foods, the supply of fresh-cut fruits and vegetables in the market has increased significantly in recent years [16]. Fresh-cut fruits and vegetables are widely welcomed by consumers because of their ready-to-eat convenience, nutritional value and health benefits. However, tissue damage from cutting accelerates the deterioration of quality, and also promotes the proliferation of microorganisms [17]. Therefore, the preservation of fresh-cut vegetables and fruits has been the focus of research in recent years. Enzymatic browning is a major problem for most fresh fruits and vegetables because of its adverse effects on their safety and sensory properties and nutritional value. As mentioned above, tyrosinase is related to the formation of brown pigment in fruits and vegetables during browning [18]. Several studies found that tannins are good inhibitors of tyrosinase [19] and are strong antioxidants [20], so tannins could represent a potential preservative.

In this study, ^13^C-NMR and RP-HPLC were used to identify and analyze part of the structure of CTs from *B. gymnorhiza*. The weight-loss rate, browning degree, polyphenol oxidase (PPO) activity, peroxidase (POD) activity, malondialdehyde (MDA) content and total phenol content (TPC) of fresh-cut lotus root were measured after treatment with CTs. The obtained knowledge can guide the development of CTs as a potential new natural antioxidant, tyrosinase inhibitor and food preservative, providing the theoretical basis for CTs to play an important role in cosmetics, food, medicine and other fields in the future. To our knowledge, this is the first time that the structure and biological activity of CTs from *B. gymnorhiza* have been studied. The preservation effect and the mechanism of *B. gymnorhiza* CTs on fresh-cut lotus root are also discussed.

## 2. Results and Discussion 

### 2.1. Total Phenolics and Condensed Tannins Contents in the Leaf of B. gymnorhiza

In this study, CTs extracted from the freeze-dried leaves of *B. gymnorhiza* and purified by chromatography over Sephadex LH-20 were used, and the standard curve was developed by the butanol–HCl method. Of 1 mg lyophilized powder of *B. gymnorhiza* leaves was dissolved into 1 mg/mL of sample for butanol-HCl method. The results showed that the leaves of *B. gymnorhiza* had high TPC (251.48 ± 0.27 mgGAE/gDW) and CTs (133.75 ± 0.04 mg/gDW).

### 2.2. ^13^C-NMR Analysis of CTs

**Figure 2:**
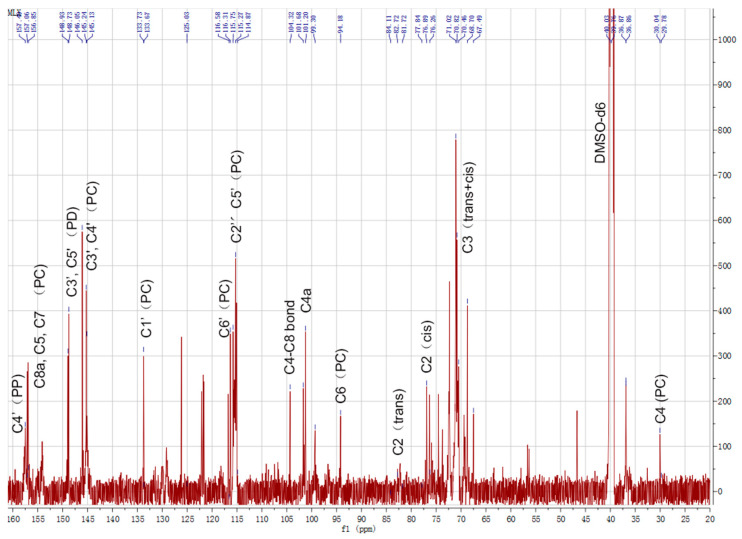
^13^C-NMR spectrum of CTs from *B. gymnorhiza*.

Structural characterization of the CTs in the leaves of *B gymnorhiza* was performed by ^13^C-NMR. Of samples 100 mg were dissolved in 500 μL DMSO-*d*_6_ and then loaded into a nuclear magnetic tube for detection. The ^13^C-NMR was used to determine the properties of CTs, including PC, the PD extension unit and the heterocyclic ring structure. The distribution of NMR signals was based on the analysis of literature data [13,21,22,23,24,25]. The ^13^C-NMR spectrum (Figure 2) showed characteristic ^13^C-peaks consistent with that of CTs with dominant PC units, and some PD units and PP units. ^13^C-NMR (151 MHz, DMSO) could detect C-related of A and B ring structures. The solvent peak was located at 40–41 ppm. When the hydroxyl group of C3 is esterified, the C4 site of the catechin or gallocatechin unit appears at the spectral peak of 23.6 ppm. However, no signal was detected by ^13^C-NMR at 23.6 ppm, which indicates an unesterified hydroxyl group carried by the PC C3. Moreover, C4 showed a high signal peak at 30.04 ppm (29.78 ppm). The C4 sites were shown to possess interflavanoid C4–C6 or C4–C8 bonds with a chemical shift at 36.87 ppm and a C3 signal occurring between 64 and 78 ppm of B rings. The 68.70 ppm peak represented the terminal structure of C3 and 71.02 ppm peak represented the *cis*–*trans* isomer signal of C3. The 77 ppm and 82.72 ppm peaks represented the *cis*–*trans* structure of C2 and, according to the peak area estimation, the *cis*-structure of C2 dominated (97%). In addition, areas between 65 and 77 ppm may contain characteristic signals of carbohydrates (glycosides) in CT extracts, which sometimes overlapped with the CT signals. These signals were weak and were hard to distinguish in the liquid ^13^C-NMR, while it was compared to the solid-phase ^13^C-NMR spectra for subsequent analysis. The 94.19 ppm signal peak represented C6. No obvious C4–C8 bond signal peak was detected at 110 ppm, though a high-strength C4–C6 bond signal was detected at around 106 ppm, indicating that CTs of *B. gymnorhiza* were composed of PCs combining C4–C6 without the C4–C8 bond. The signals at 114.87 and 115.27 ppm were assigned to the C2′ and C5′ of PC units, respectively. The signals at around 116.58 ppm were due to the C6′ sites. The 145.13/145.24 and 146.05 ppm signal peaks represented PC C3′, C4′ and PD C3′, C5′, respectively. Resonances around 146 ppm corresponding to the C3′ and C5′ B ring sites of PD were very weak in the spectrum. The relative ratio of PC/PD, which was calculated based on the area of the single peak of 145 ppm and 146 ppm, was 1.78. The peak at 156.85–157.49 ppm represented C8a, C5 and C7 for PC and C4′ of PP. 

### 2.3. RP-HPLC Analysis 

**Figure 3:**
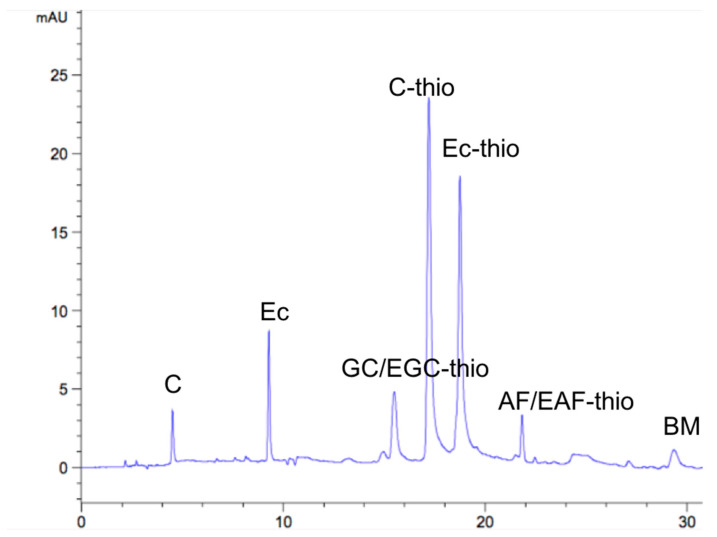
HPLC spectrum of CTs from *B. gymnorhiza*.

In order to characterize the structural information of *B. gymnorhiza* CTs, sulfur hydrolysis was combined with HPLC chromatographic analysis as a tool for evaluating CTs structural units. With benzene thiol as the nucleophile, the terminal unit was released as a free monomer, while the external unit was formed as a benzene thiol ether plus a monomer in the sulfurization reaction [26]. After CTs were degraded under acidic conditions, the results obtained by HPLC were presented, as shown in Figure 3. Combined with retention time (Table 1), these results revealed that the basic constituents of CTs in the leaves were catechin/epigallocatechin (C/EC), epigallocatechin/epigallocatechin benzyl sulfide (GC/EGC) and a small amount of afzelechin/epafodoxine (AF/EAF), and the corresponding structures were PC, PD and PP, which were consistent with the ^13^C-NMR spectra. In addition, combined with the peak area, the average mean polymerization degree (mDP) of *B. gymnorhiza* CTs was 4.85. The above results strongly suggested that the main component of CT_S_ in *B. gymnorhiza* leaves was PC, which was composed of catechin units.

In terms of structure identification of tannins, only the types of main components and the structures of basic units were determined. The degree of polymerization of tannins and the specific proportion of each component were not analyzed. In terms of isolation and purification technology, it is necessary to combine various isolation technologies to obtain as many monomer substances as possible for subsequent in-depth experiments. In addition, based on the existing HPLC and ^13^C-NMR data, more experiments could be used to further determine the chemical displacement and structures of important CTs compounds. These data could contribute to further studies of the structures of CTs and their interactions with other substances.

### 2.4. Inhibition Kinetics of Mushroom Tyrosinase 

**Figure 4:**
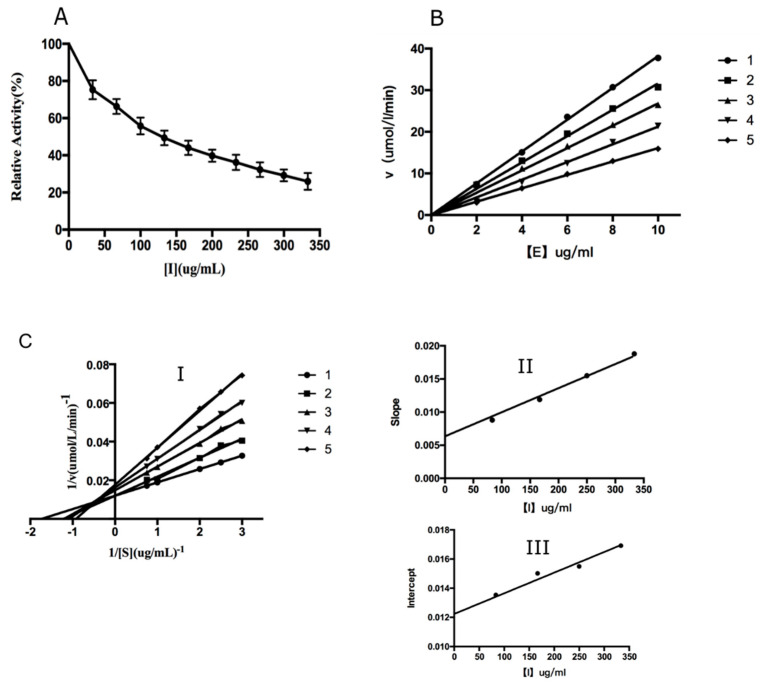
(**A**). The inhibitory effect activity of CTs. (**B**). Inhibitory mechanism of CTs. (**C**). Inhibitory type of condensed tannins. Curves 1–5 indicate that the final concentrations of CTs were 0, 83.30, 166.67, 250.00, and 333.33 μg/mL, respectively.

The effects of CTs in leaves of *B. gymnorhiza* on mushroom tyrosinase activity were studied and the results are shown in Figure 4. With increasing concentrations of CTs, the activity of the enzyme was observably reduced. The IC_50_ value of CTs was estimated to be 123.90 ± 0.140 μg/mL (Figure 4A). The data showed that the residual enzyme activity was plotted against the enzyme quantity to obtain a straight line through the origin, and the reversible effect of the inhibitor on TYR was judged [27] (Figure 4B). The enzyme concentration was fixed and the substrate l-DOPA concentrations were changed, allowing determination of the original data of the enzyme reaction rate using a Lineweaver–Burk plot and obtainment of the inhibition type (Figure 4C). Thus, it was judged that the effector affected the Vm and Km value at the same time. With the increase in inhibitor concentration, Km value increased while Vm value decreased, so its inhibition type was mixed (Figure 4C-I). The inhibition constants (K_I_ and K_IS_) were obtained by the conic curve of 1/Vm or Km/Vm to the inhibitor concentration (Figure 4C-II,III). The K_I_ and K_IS_ obtained by the straight line were 160 μg/mL and 122 μg/mL, respectively. According to existing literature, CTs in plants can chelate with copper ions in the active center of TYR through the hydroxyl group on the basic unit structure of a B ring, resulting in conformational changes of enzymes, thus reducing the activity of TYR [28]. Proanthocyanidin is an effective tyrosinase inhibitor [29,30], and the main component in CTs of *B. gymnorhiza* is proanthocyanidin. Proanthocyanidin molecules may bind to the copper ion active center of TYR. The concentration of free enzyme in the reaction system decreased and the conformational changes of the enzyme inhibited the activity of the enzyme [31]. It is also possible that the binding of the target substance to the reactant caused competition, reducing the probability of the enzyme binding to the substrate and thus reducing the initial reaction quantity [32]. Based on the structural characteristics and tyrosinase analysis, we hypothesized that CTs could inhibit tyrosinase due to its similar structure with l-DOPA, the substrate. At present, tannins extracted from plants such as *Dioscorea cirrhosa* [33], cherry branch [34], macroalgae [35], spruce [36] and other plants were studied for their antioxidation, anti-TYR and antibacterial properties. However, from the perspective of application, due to the strong antioxidant properties of plant tannins, great development potential exists in whitening agents, medicine, food and other aspects as TYR inhibitors.

### 2.5. Antioxidant Activity of CTs

**Figure 5:**
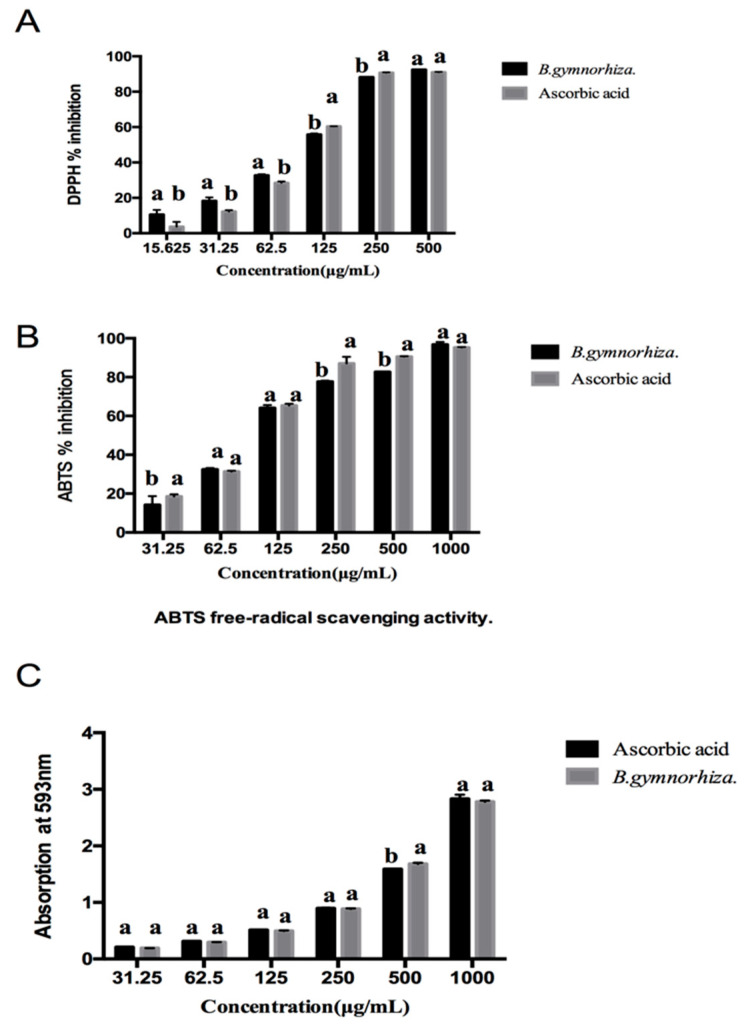
Antioxidant analysis of condensed tannins from *B. gymnorhiza*. (**A**) DPPH assay, (**B**) ABTS assay and (**C**) FRAP assay.

DPPH, ABTS and FRAP were used to measure the antioxidant activity of CTs in the leaves of *B. gymnorhiza*. These results are shown in Figure 5. At the same concentration, the scavenging rate of DPPH radicals by CTs was similar to that of Vc. The IC_50_ values of CTs and Vc were 88.81 ± 0.135 μg/mL and 97.65 ± 0.153 μg/mL, respectively. The scavenging capacity of CTs on ABTS radicals was enhanced with the increase in the concentration of the substance and showed a dose effect. The IC_50_ value of CTs was 105.03 ± 0.130 g/mL, higher than that of Vc (88.84 ± 0.192 g/mL) under the same conditions. In addition, when the drug concentration reached 1000 g/mL, the scavenging rates of CTs and Vc for ABTS free radicals reached 96.83% and 95.28%, respectively. The FRAP antioxidant value of CTs was 1052.27 ± 4.17 mg AAE/g, as shown in Table 2. According to literature reports, the antioxidant capacity of CTs is different due to the different structure of the B ring [37]. The hydroxyl groups on the B rings of gallocatechin, catechin and afzelechin are used as hydrogen donors. Oxygen was reduced to low active tri-line oxygen in a catalytic reaction to reduce the probability of oxygen free-radical generation or to terminate the reaction. The scavenging capacity of free radicals was increased with the increasing number of flavan-3-alcohol units and hydroxyl groups in B ring, resulting in the strong antioxidant capacity of CTs shown in *B. gymnorhiza*.

### 2.6. Evaluation of CTs Treatment on Fresh-Cut Lotus Root 

**Figure 6:**
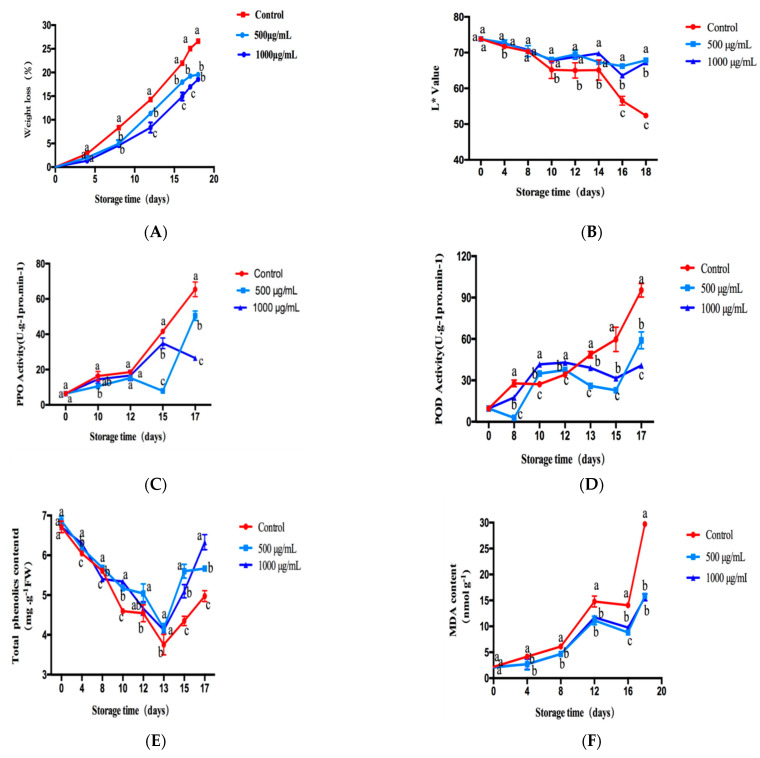
The preservation effect of CTs on fresh-cut lotus root. The lotus root was treated with different concentrations of CTs. Then different effects were measured during the storage at 4 °C for 17 days. (**A**) The weight-loss ratio. (**B**) The L* value. (**C**) The activities of polyphenol oxidase (PPO). (**D**) The activities of peroxidase (POD). (**E**) The total phenol content (TPC). (**F**) The malondialdehyde (MDA) content.

We evaluated the preservation effect of CTs by detecting the weight-loss rate, browning degree and total phenol content of fresh-cut lotus root; these results are shown in Figure 6.

#### 2.6.1. Effect of CTs on the Weight-Loss Ratio of Fresh-Cut Lotus Root 

The weight-loss rate can reflect the freshness of fruits and vegetables. The weight-loss ratio of fresh-cut lotus root treated with the different concentrations of CTs was investigated. As shown in Figure 6A, with the extension of storage time, the weight loss rate of freshly cut lotus root gradually increased. The lower the weight-loss rate, the less the nutrient loss of the lotus root. The results showed that on the 18th day, the weight-loss rate of fresh-cut lotus root in the control group was 26%, while that in the experimental group was 18%. Therefore, we speculated that the reason for the reduced weight-loss rate of fresh lotus root was that CTs inhibited respiration by reducing the activity of the respiratory chain enzyme, thus reducing the water loss of fresh lotus root. Moreover, statistical analysis suggested that the weight-loss ratio of fresh-cut lotus root treated with 500 ug/mL and 1 mg/mL of CTs was significantly (*p* < 0.05) lower than that of the control group from day 4 to day 17 of the storage period. These results demonstrated that CTs could significantly reduce the weight loss of fresh-cut lotus root.

#### 2.6.2. Effect of CTs on L* of Fresh-Cut Lotus Root

Uniform slices of fresh lotus root were randomly selected. Due to mechanical damage, the cutting surface was easily oxidized when exposed to air and the surface appeared dark brown. The browning of fresh-cut lotus root was evaluated by measuring the color difference (L*) on its surface. The measured results are shown in Figure 6B. The larger the L* value was, the whiter the skin was and the lighter the browning became. On day 0, there was no significant difference in L* between the experimental group and the control group, indicating that the initial state of the experimental group was the same as the control group. With the increasing storage time, the L* of fresh-cut lotus root in the treatment group and control group became smaller, indicating that the browning was more serious than before, which was mainly caused by enzymatic browning. There were significant differences in L* value between the CT treatment group and control group on day 4 and day 8, respectively. About day 10–18, the browning was significantly inhibited compared to the control group, though there was no significant concentration-dependent effect between the experimental groups.

#### 2.6.3. Effect of CT Treatment on Activities of PPO and POD in Fresh-Cut Lotus Root 

PPO and POD are the most important enzymes for enzymatic browning. The higher the activity of PPO and POD, the more serious and obvious the browning. In this research, the activities of PPO and POD in fresh-cut lotus root treated with different concentrations of CTs were studied. As shown in Figure 6C, the PPO activity of CT-treated sections was significantly lower than that of the control group (*p* < 0.05). The PPO activity in the control fresh-cut lotus root showed an increasing trend from day 10 to day 17 and was significantly higher than the experimental group, further indicating that CTs could inhibit PPO activity and delay the appearance of browning. The activities of POD in fresh-cut lotus root treated with different concentrations of CTs are shown in Figure 6D. After eight days of storage, the POD activity of CT-treated sections was significantly lower than the control (*p* < 0.05). The POD activity in the treatment group was relatively stable on days 10–12, while the POD activity in the control group showed an obvious upward trend. The POD activity increased rapidly until the end of storage time. Moreover, during the storage period, the POD activity in lotus root slices in the control group was always higher.

#### 2.6.4. Effect of CTs Treatment on TPC and MDA Content in Fresh-Cut Lotus Root 

As shown in Figure 6E, in general, the total phenol content (TPC) in the control group and the CT-treated flakes decreased rapidly during storage and then increased. The total phenol content in the control group decreased faster and rose more slowly than that in the treatment group. The average value of the total phenol content in the lotus root slices treated by CTs on day 17 was about 27% higher than the control group (*p* < 0.05).

The MDA accumulation rate represented the free-radical scavenging capacity of tissue. Figure 6F shows that during the whole storage period, MDA content in the control groups increased by 92%. However, CT treatment delayed the MDA accumulation. After CT treatment, MDA content was 15.59 nmol g^−1^ FW on the 18th day, while it was about 29.72 nmol g^−1^ FW in the control group, which was significantly higher than that in the treatment group (*p* < 0.05), further indicating that CTs possess a certain degree of tissue protection ability for fresh-cut lotus root.

Fresh-cut fruits and vegetables produce tissue damage in the process of peeling and slicing, causing both enzymatic and non-enzymatic browning. The browning degree or fresh-keeping effect was mainly evaluated by change in appearance, weight-loss rate, browning degree (L* value), PPO, POD, total phenol and MDA content. The shelf-life of fresh-cut fruits and vegetables was mainly caused by surface browning, which was closely related to phenolic metabolic enzymes. For example, the combination of POD and PPO and substrate leads to the oxidation of phenols. The reason for the oxidation of phenolic substances may be that phenolic substances are distributed in vacuoles, while PPO and POD are located in the cell wall, cell membrane and cytoplasm to prevent reaction between the substrate and enzyme and avoid enzymatic browning of tissues. Due to the mechanical damage of fresh-cut lotus root, the regional distribution is destroyed, which leads to the migration of phenolic substances and full contact with PPO and POD, causing local tissue damage. Through the migration of damage signals to adjacent normal tissues, oxidative browning and the production of secondary metabolites and other physiological reactions are initiated, which are controlled by a complex cascade network. Although the freshness preservation indexes of tannins on fresh-cut lotus root were tested and demonstrated as far as possible, it is still necessary to combine LOX, APX, PAL, CAT and other indexes to evaluate the freshness preservation effect. In addition, it is better to study the transcriptional level or gene level of related enzymes, which could more convincingly elucidate the effect of tannins on the preservation of fresh-cut lotus root.

## 3. Materials and Methods

### 3.1. Chemicals and Plant Material

Dimethylsulfoxide-*d*_6_ (DMSO-*d*_6_), l-tyrosine, l-3,4-dihydroxyphenylalanine (l-DOPA), mushroom tyrosinase (EC 1.14.18.1), ascorbic acid (Vc), tripyridyltriazine (TPTZ), 2,2-diphenyl-1-picrylhydrazyl (DPPH) and 2,2′-azino-bis (3-ethylbenzo-thiazoline-6-sulfonic acid diammonium salt) (ABTS) were obtained from Sigma-Aldrich (St Louis, MO, USA). All analytical grade solvents and HPLC-grade dichloromethane, acetonitrile (CHCN), methanol, HPLC-grade CH_3_CN and trifluoroacetic acid (TFA) were obtained from Sinopharm (Sinopharm, Shanghai, China).

Green leaves of *B. gymnorhiza* were collected from Hangjiang mangrove wetland park (Yunxiao country, Zhangzhou city, Fujian province) during March 2018. The leaves were separated, washed and freeze-dried immediately in the laboratory. These materials were ground to powders through a 40-mesh sieve after crushing the samples for 2 min and freeze-drying. The fine powders were then frozen at −20 °C for later use.

### 3.2. Extraction and Purification of the CTs

The method was according to Deng, Y.T et al. [38]. Leaf powders (10 g) were ultrasonically extracted 3 times (30 min at a time) with 300 mL of acetone-water (70%, *v*/*v*) at 25 °C, and acetone was removed by rotary evaporation under vacuum at 40 °C. The aqueous solution was firstly extracted by petroleum ether (3 times, 300 mL) to remove the fat-soluble substances and nonpolar components. Then, the aqueous solution was extracted by ethyl acetate to remove low molecular phenolic substances, and the crude extract was obtained by rotary evaporation and freeze-drying. To obtain the CTs, a 50% (*v*/*v*) methanol aqueous solution was used to dissolve the crude extract. It was then separated by Sephadex LH-20 column chromatography (Pharmacia Biotech, Uppsala, Sweden), which was then eluted with methanol–water (50%, *v*/*v*) to remove sugars, glycosides and monomeric polyphenols and eluted with acetone–water (70%, *v*/*v*). Rotary evaporation was then used to remove the methanol and acetone. The fractions of 70% acetone–water were retained to be freeze-dried as purified CT powders (376 mg). The powders were stored at −20 °C for later use. 

### 3.3. Determination of Total Phenolics and Proanthocyanidins Content 

Total phenol content was determined according to the Folin-Ciocalteu method [39], with gallic acid used as the standard curve. A total of 0.2 mL of the standard/sample, 300 µL of ddH_2_O, 500 µL of Folin-Ciocalteu (FC) reagent and 2.5 mL of 20% Na_2_CO_3_ were mixed and reacted for 50 min in a dark place. Then, optical density (OD) values were measured at 760 nm. TPC was measured as mgGAE/gDW. Determination of CTs was carried out using the butanol-HCl method [40]. The samples were prepared with a series of concentration gradients and thoroughly mixed according to the proportion of the sample solution (*n*-butanol/HCl solution (95:5, *v*/*v*) = 1:6). After boiling in a water bath for 60 min, the purified CTs were used as the standard. The absorbance values were measured at the wavelength of 550 nm. All experiments were conducted in triplicate.

### 3.4. ^13^C-NMR Analysis

Analysis followed the literature method [41], a Varian Mercury-600 spectrometer (Palo Alto, CA, USA) was used for structural ^13^C-NMR analysis of CTs at a frequency of 150 MHz. The CTs were dissolved with dimethylsulfoxide-*d*_6_. Of the 500 µL sample solution was placed in a 5 mm NMR test tube and ^13^C-NMR spectrum data were recorded.

### 3.5. Performance Liquid Chromatography Analysis

Analysis followed the literature-provided experimental methods [35], a sample solution of 5 mg/mL was accurately weighed before preprocessing by liquid chromatography. The solution of the CTs, hydrochloric acid–methanol solution (3.3%) and benzyl mercaptan-methanol solution (5%) were mixed and incubated at 40 °C for 30 min before filtering with a 0.45 μm filter membrane. The HPLC used for the CTs detection was the Agilent 1200 system (Agilent, Santa Clara, CA, USA) equipped with a quaternary pump and diode array detector. Before loading, ultrasonic degassing was performed for 30 min to clean the pipeline passage and flow column. The column for analysis was a 250 mm × 4.6 mm i.d., 5 μm, LiChrospher 100 RP-18, with a 4 mm × 4 mm i.d. guard column of the same material (Elite, Dalian, China). The sample loading flow rate was 1 mL/min. The linear gradient was 0–45 min, 12–80% CH_3_CN, 45–50 min and 80–12% CH_3_CN. The mobile phase was 0.5% TFA and the column temperature was 25 °C. UV spectrum detection wavelengths were 254 nm and 280 nm, respectively. The data results were analyzed by referring to the reported literature [42]. We calculated the mean of DP using the following equation: mean DP = (total peak area of the extender units)/(total peak area of the terminal units) + 1.

### 3.6. Tyrosinase Inhibitory Activities 

l-DOPA (0.5 mM) was used as the substrate in 3 mL aliquots, and the final concentration of mushroom tyrosinase was 3.33 g/mL [43]. The absorbance value at the wavelength 475 nm was monitored at a constant temperature of 30 °C by Thermo Scientific Multiskan Go. The slope of the reaction curve represented diphenolase activity. The relative residual enzyme activity of diphenolase was used to plot the sample concentration and calculate 50% tyrosinase activity (IC_50_), which was used to plot the enzyme concentration, and the inhibition mechanism of CTs on diphenolase was determined according to the characteristics of linear intersection [44]. By using a Lineweaver-Burk double reciprocal graph, the Michaelis-Menten constant (km) and maximum velocity (Vm) values were calculated [27], to determine the inhibition type of plant tannins on diphenolase.

### 3.7. DPPH Assay

The ability to scavenge DPPH on the CTs was detected according to the method reported by Adeline Vignault et al. [45]. The vitamin C solution with the same concentration gradient (0, 15.625, 31.25, 62.50, 125.00, 250.00, and 500 μg/mL) was used as the positive control. DPPH solution configuration consisted of 3.5 mg DPPH powder weighed and dissolved in methanol at OD517 nm, adjusting the absorbance to 0.7–0.8 in the dark and setting aside. The solutions of the experimental and control groups with different concentrations were added to different tubes and then thoroughly mixed with the DPPH solution at a ratio of 1:19 respectively. After the mixture was shaken vigorously and incubated in the dark (25 °C, 30 min), the absorbance at 517 nm was read (A1). A0 is the absorbance value of DPPH-methanol solution. The data were analyzed using the formula DPPH% inhibition = [(A0 − A1)/A0] × 100%.

### 3.8. Scavenging Activity on ABTS Radical 

According to the method by Abu Zarin, M et al. [46], the ABTS radical mother liquor was scavenged and configured as 70 mmol/L ABTS and reacted with 50 mmol/L potassium persulfate in 1:1 (*v*/*v*) in the dark for 16 h. The ABTS radical mother liquor was diluted with 80% ethanol solution until OD 734 nm = 0.65–0.75 then used as the working liquid. The 10 µL sample solution (31.25–1000 μg/mL) and 190 µL diluted ABTS solution were thoroughly mixed, then added to the 96-well plate. The reaction was conducted at room temperature and in dark for 6 min. The OD value was measured at 734 nm with ascorbic acid (Vc) as a positive control and 80% ethanol diluted working liquor as a blank control.

### 3.9. FRAP Assay

The ferric ion reducing antioxidant power (FRAP) assay was used to evaluate the CTs by the following method [47]: 100 μL samples of different concentrations (31.25–1000 μg/mL) were taken and mixed with 500 μL of PBS (0.2 m, pH = 6.6) and 0.5 mL of 10 mg/mL potassium ferricyanide. The remaining FRAP reagent (2 mL) was added before cooling for 20 min. After mixing, stand the solution for 5 min. The absorbance was measured at the wavelength of 593 nm. Vc solution with corresponding concentration gradient was used as the positive control group. 

### 3.10. Measurement of Fresh-Cut Lotus Root Storage Quality

Fresh-cut lotus roots without large area damage were purchased from a market in Xiamen, China. After being rinsed and peeled, evenly cut lotus root chunks of the same mass (mass error ±0.5 g) were immediately soaked in aqueous tannin solution of different concentrations for 20–30 min and drained at room temperature or with absorbent paper. All treated lotus root slices were put into plastic bags, marked, and stored in a 4 °C refrigerator until further analysis. During a period of storage, the weight loss, the whiteness value (L* value), PPO, POD, TPC, and MDA were measured at intervals of 2 days in triplicate to evaluate the storage quality of fresh-cut lotus root.

In particular, L* value was measured using the reference of the ADCI-60-C colorimeter [48]. The larger the value of L*, the whiter the color of the sample. PPO and POD activities were extracted and assayed by the reported method [49,50]. The results were expressed as units of enzyme/g protein. TPC was determined according to the previous research and the results were expressed in units of gallic acid equivalent mass, based on fresh weight, as mg g^−1^ FW (fresh weight). Specific measurement system and data analysis were conducted by referring to the specification of plant malondialdehyde (MDA) test box of Nanjing Jiancheng institute of biological engineering.

### 3.11. Data Analysis

The values were expressed as mean ± standard deviation (n = 3), and the significance analysis was marked with letters (a, b, c, etc.), which showed significant differences (*p* < 0.05). The data analysis was performed by the post-event multiple comparison of Duncan’s hypothesis using the one-way ANOVA test. All experiments were repeated three times.

## 4. Conclusions

In conclusion, the leaves CTs of *B. gymnorhiza* were mixtures of B-type PC, PP and PD. CTs are connected through the C4-C6 bond between flavantriols and the mDP is about five, belonging to the oligomer category, without containing esterified procyanidins. Moreover, this study demonstrated that CTs showed remarkable antityrosinase and antioxidant capacities. The results of DPPH, ABTS and FRAP revealed that CTs were efficient, reversible and mixed competitive types of tyrosinase inhibitors.

In addition, the preservation assays manifested that CTs could inhibit the activities of PPO and POD, and decrease the oxidation of total phenolics and MDA in fresh-cut lotus root, thereby preventing the browning of fresh-cut lotus root. The biological potentials of other constituents in *B. gymnorhiza* should be investigated in future studies.

## Figures and Tables

**Table 1:** Retention time obtained by RP-HPLC in CTs from *B. gymnorhiza.* Unit: min.

Flavan-3-ol	The End of the Unit		Extended Unit Adduct				Solvent
	C	EC	GC/EGC	C	EC	AF/EAF	BM
Standards	4.4	9.5	15.4	17.0	18.4	21.5	27.5
CTs	4.55	9.35	15.53	17.25	18.64	21.89	29.63

**Table 2:** Antioxidant activities of CTs from *B. gymnorhiza*.

Samples	DPPH (IC_50_ μg/mL)	ABTS (IC_50_ μg/mL)	FRAP (mg AAE/g)
*B. gymnorhiza*	88.81 ± 0.135 ^b^	105.03 ± 0.134 ^a^	1052.27 ± 4.170
Ascorbic acid	97.65 ± 0.153 ^a^	89.55 ± 0.274 ^b^	—

## Data Availability

Data presented are available in the manuscript.

## Sample Availability

Samples of the compounds Condensed Tannins from *Bruguiera gymnorhiza* (L.) Lam are available from the authors.

## Abbreviations

CTs	condensed tannins
AAE	ascorbic acid equivalent
AF/EAF	afzelechin/epafodoxine
ABTS	2,2′-azino-bis(3-ethylbenzo-thiazoline-6-sulfonic acid diammonium salt)
C/EC	catechin/epicatechin
DPPH	2,2-diphenyl-1-picrylhydrazyl
FRAP	ferric ion reducing antioxidant power
IC_50_	50% inhibiting concentration
UV	ultraviolet
^13^C-NMR	^13^C nuclear magnetic resonance
Km	Michaelis-Menten constant
l-DOPA	l-3,4-dihydroxyphenylalanine
PBS	phosphate buffer
PC	procyanidin
PD	prodelphinidins
PP	propelargonidin
TFA	trifluoroacetate acid
PPO	polyphenol oxidase
POD	peroxidase
MDA	malondialdehyde
TPC	total phenolics content
Vc	ascorbic acid
Vm	maximum velocity
L*	whiteness value
mDP	mean polymerization degree
OD	optical density
GAE	gallic acid concentration
DW	dry weight
LOX	lipoxygenase
APX	ascorbate peroxidase
PAL	phenylalnine ammonialyase
CAT	catalase
